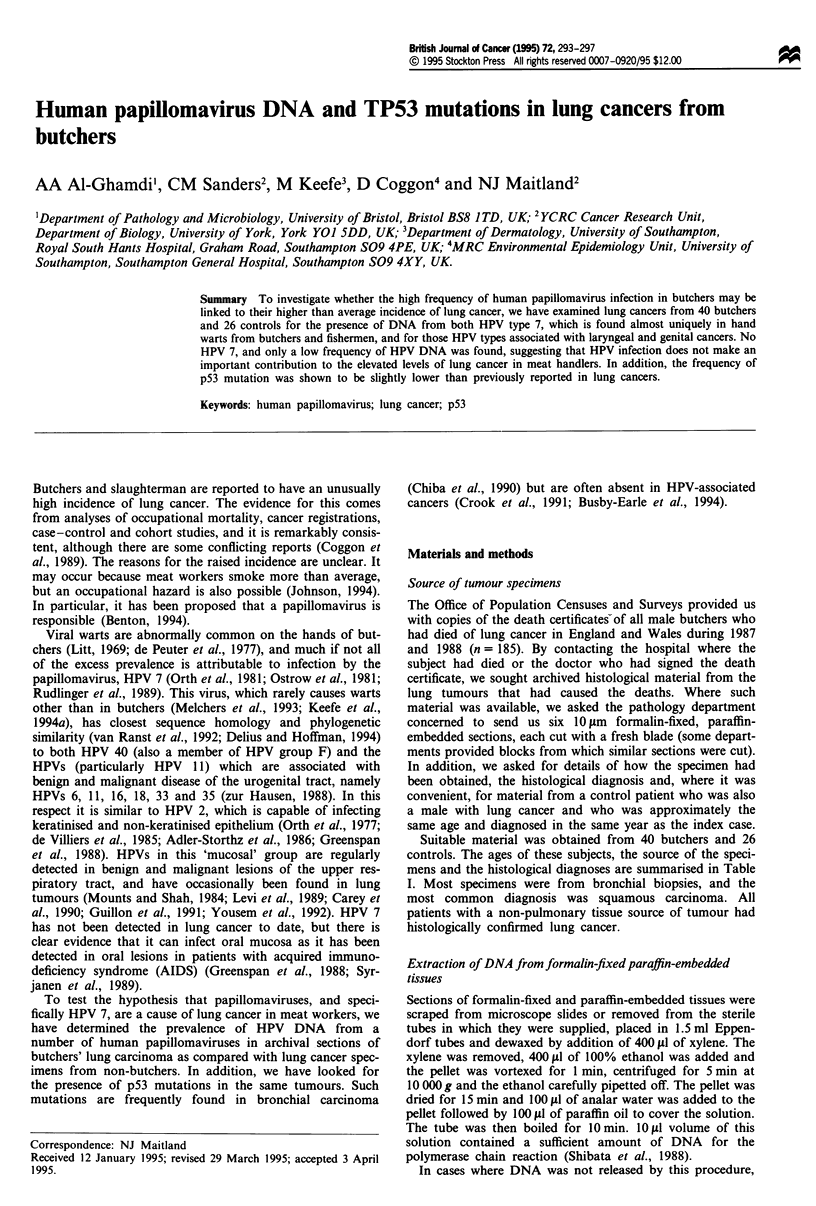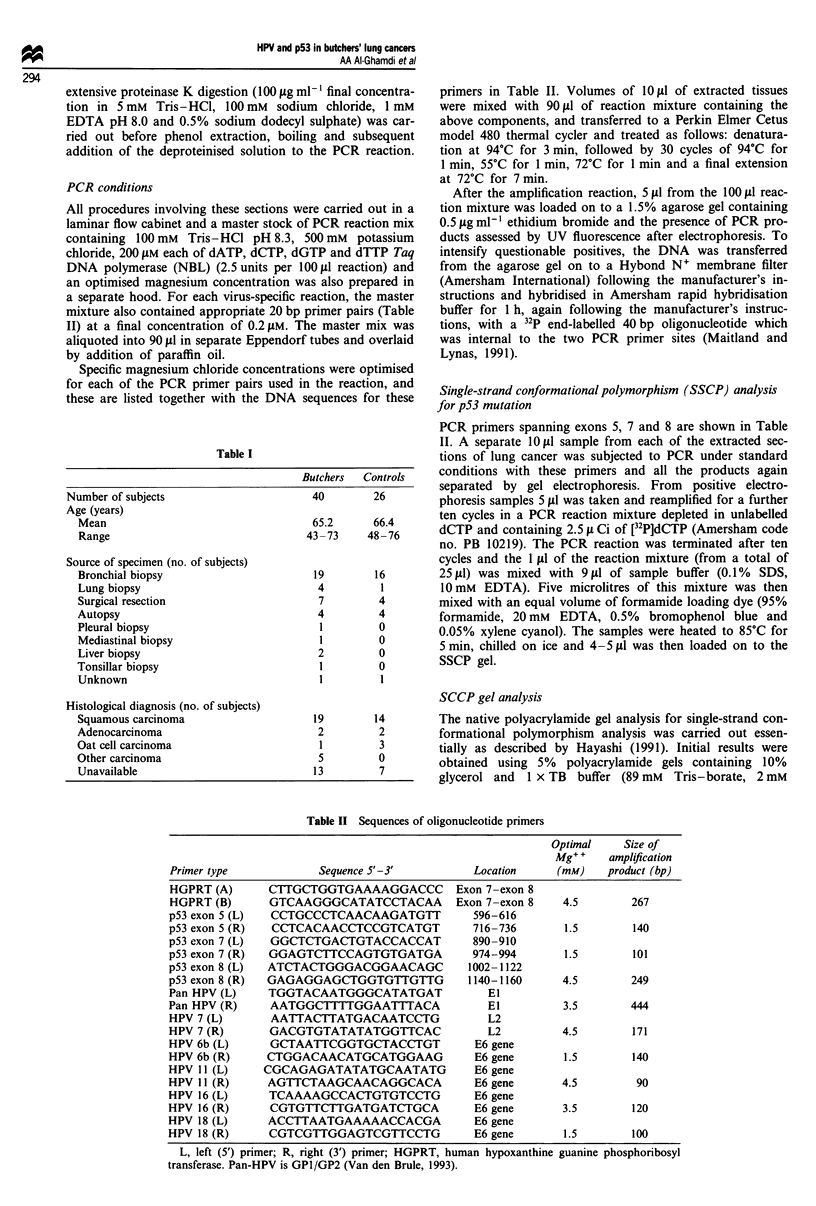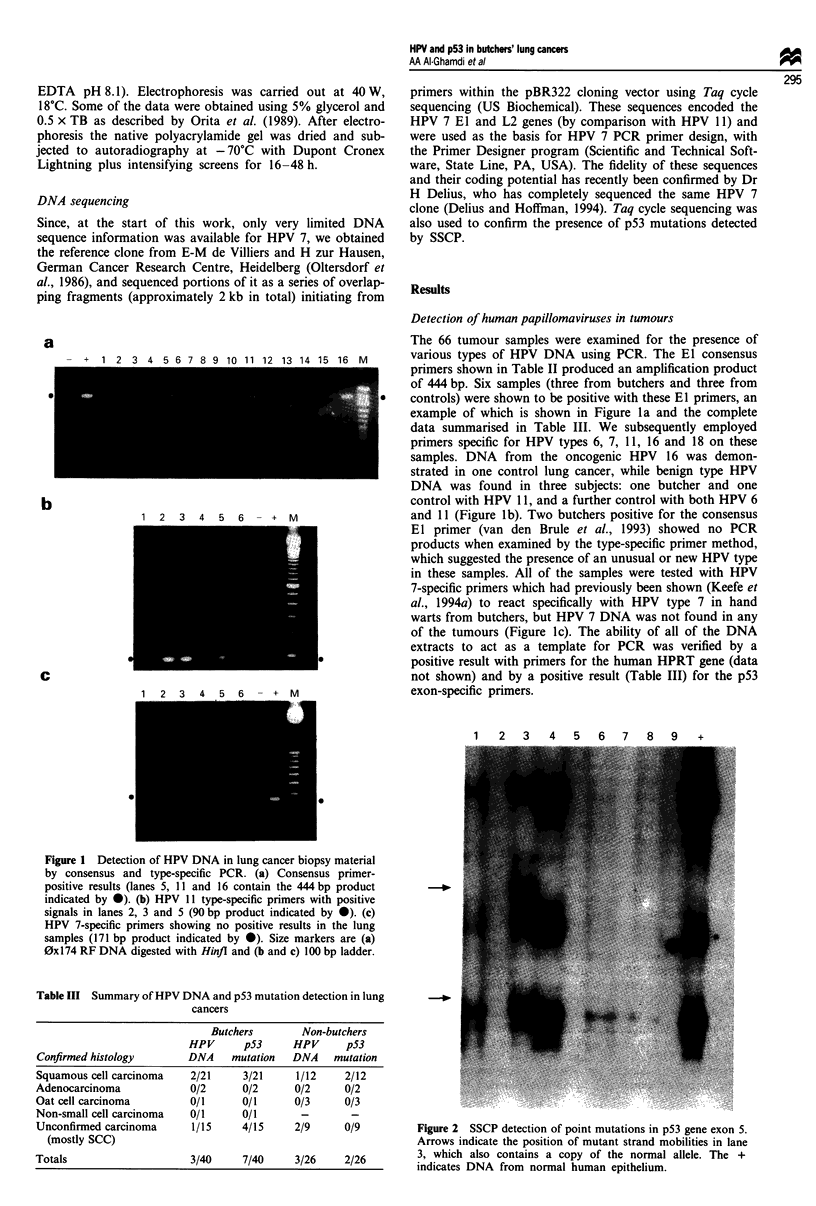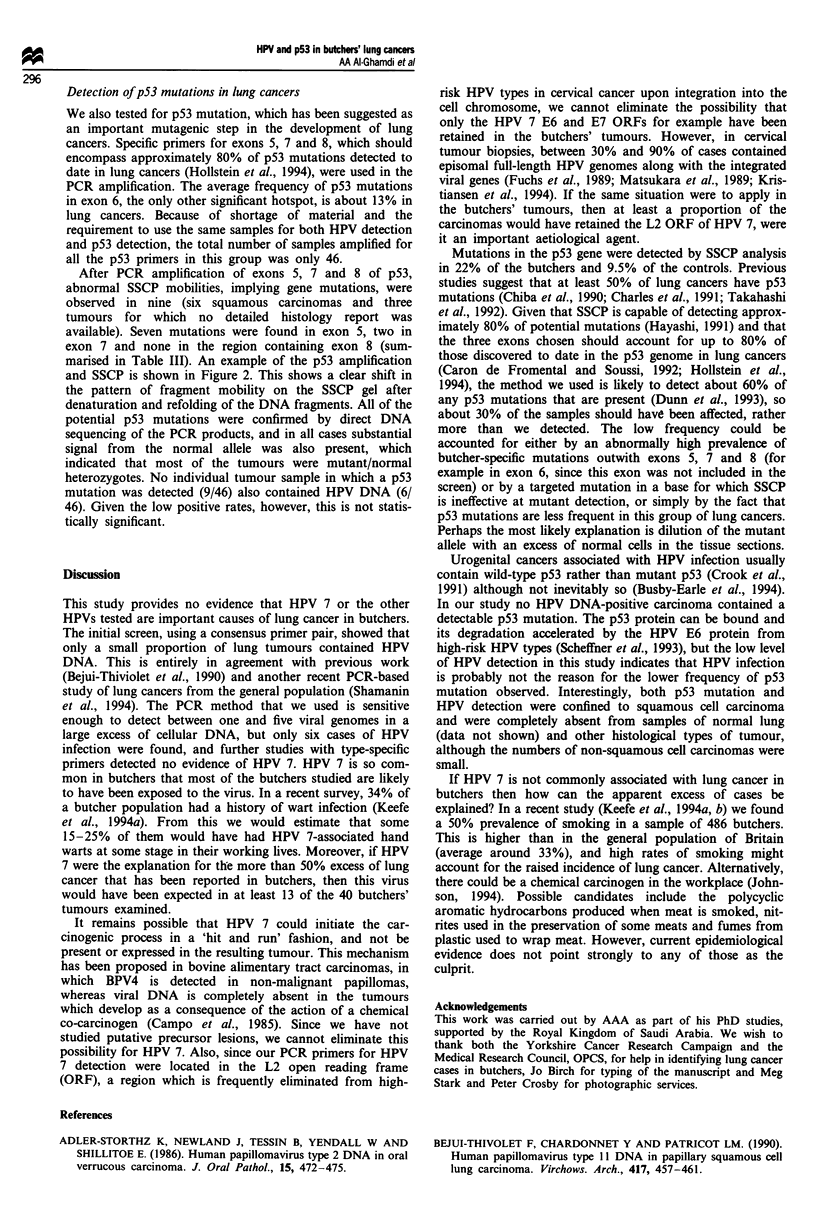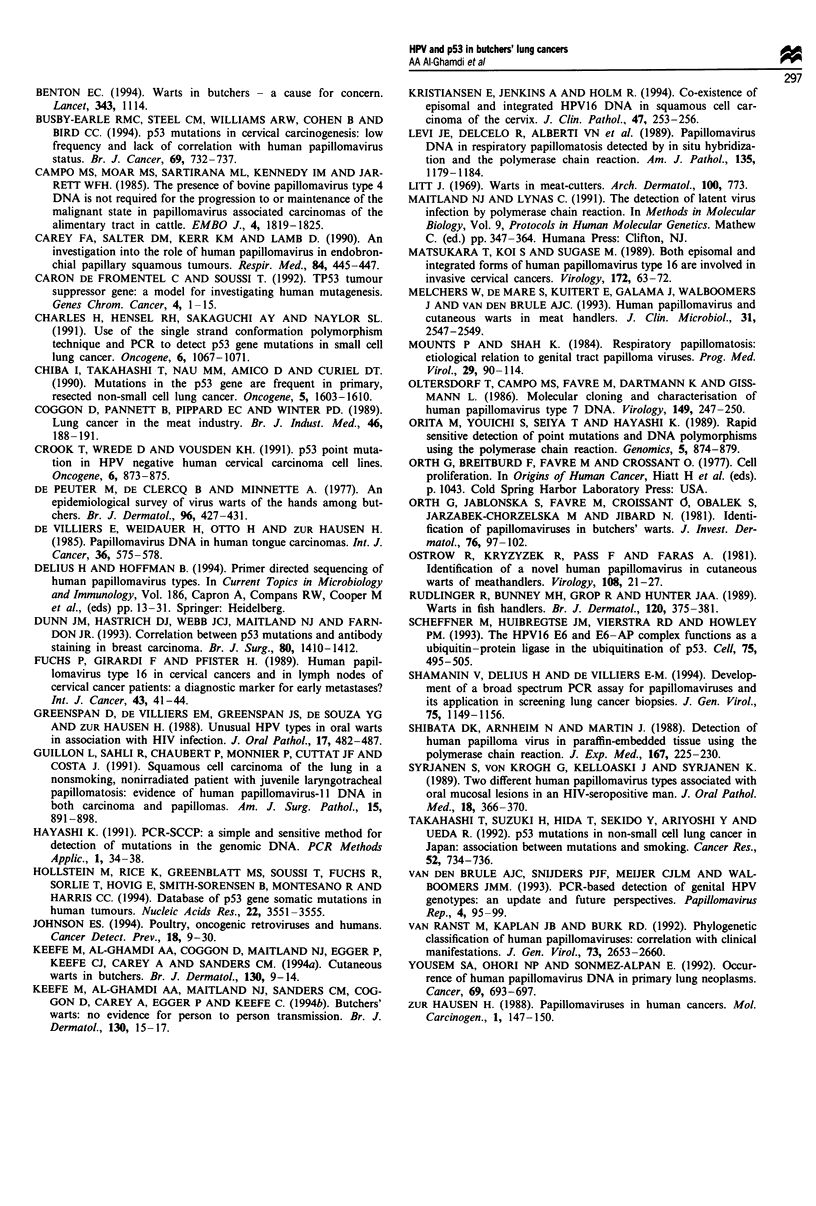# Human papillomavirus DNA and TP53 mutations in lung cancers from butchers.

**DOI:** 10.1038/bjc.1995.327

**Published:** 1995-08

**Authors:** A. A. al-Ghamdi, C. M. Sanders, M. Keefe, D. Coggon, N. J. Maitland

**Affiliations:** Department of Pathology and Microbiology, University of Bristol, UK.

## Abstract

**Images:**


British Journal of Cancer (1995) 72, 293-297
© 1995 Stockton Press All rights reserved 0007-0920/95 $12.00

### Human papillomavirus DNA and TP53 mutations in lung cancers from butchers

AA Al-Ghamdi[1], CM Sanders[2], M Keefe[3], D Coggon[4] and NJ Maitland[2]

[1]*Department of Pathology and Microbiology, University of Bristol, Bristol BS8 1TD, UK;* [2]*YCRC Cancer Research Unit, Department of Biology, University of York, York YO1 5DD, UK;* [3]*Department of Dermatology, University of Southampton, Royal South Hants Hospital, Graham Road, Southampton SO9 4PE, UK;* [4]*MRC Environmental Epidemiology Unit, University of Southampton, Southampton General Hospital, Southampton SO9 4XY, UK.*

**Summary** To investigate whether the high frequency of human papillomavirus infection in butchers may be linked to their higher than average incidence of lung cancer, we have examined lung cancers from 40 butchers and 26 controls for the presence of DNA from both HPV type 7, which is found almost uniquely in hand warts from butchers and fishermen, and for those HPV types associated with laryngeal and genital cancers. No HPV 7, and only a low frequency of HPV DNA was found, suggesting that HPV infection does not make an important contribution to the elevated levels of lung cancer in meat handlers. In addition, the frequency of p53 mutation was shown to be slightly lower than previously reported in lung cancers.

**Keywords**: human papillomavirus; lung cancer; p53

Butchers and slaughterman are reported to have an unusually high incidence of lung cancer. The evidence for this comes from analyses of occupational mortality, cancer registrations, case-control and cohort studies, and it is remarkably consistent, although there are some conflicting reports (Coggon *et al.*, 1989). The reasons for the raised incidence are unclear. It may occur because meat workers smoke more than average, but an occupational hazard is also possible (Johnson, 1994). In particular, it has been proposed that a papillomavirus is responsible (Benton, 1994).

Viral warts are abnormally common on the hands of butchers (Litt, 1969; de Peuter *et al.*, 1977), and much if not all of the excess prevalence is attributable to infection by the papillomavirus, HPV 7 (Orth *et al.*, 1981; Ostrow *et al.*, 1981; Rudlinger *et al.*, 1989). This virus, which rarely causes warts other than in butchers (Melchers *et al.*, 1993; Keefe *et al.*, 1994*a*), has closest sequence homology and phylogenetic similarity (van Ranst *et al.*, 1992; Delius and Hoffman, 1994) to both HPV 40 (also a member of HPV group F) and the HPVs (particularly HPV 11) which are associated with benign and malignant disease of the urogenital tract, namely HPVs 6, 11, 16, 18, 33 and 35 (zur Hausen, 1988). In this respect it is similar to HPV 2, which is capable of infecting keratinised and non-keratinised epithelium (Orth *et al.*, 1977; de Villiers *et al.*, 1985; Adler-Storthz *et al.*, 1986; Greenspan *et al.*, 1988). HPVs in this 'mucosal' group are regularly detected in benign and malignant lesions of the upper respiratory tract, and have occasionally been found in lung tumours (Mounts and Shah, 1984; Levi *et al.*, 1989; Carey *et al.*, 1990; Guillon *et al.*, 1991; Yousem *et al.*, 1992). HPV 7 has not been detected in lung cancer to date, but there is clear evidence that it can infect oral mucosa as it has been detected in oral lesions in patients with acquired immunodeficiency syndrome (AIDS) (Greenspan *et al.*, 1988; Syrjanen *et al.*, 1989).

To test the hypothesis that papillomaviruses, and specifically HPV 7, are a cause of lung cancer in meat workers, we have determined the prevalence of HPV DNA from a number of human papillomaviruses in archival sections of butchers' lung carcinoma as compared with lung cancer specimens from non-butchers. In addition, we have looked for the presence of p53 mutations in the same tumours. Such mutations are frequently found in bronchial carcinoma (Chiba *et al.*, 1990) but are often absent in HPV-associated cancers (Crook *et al.*, 1991; Busby-Earle *et al.*, 1994).

Correspondence: NJ Maitland
Received 12 January 1995; revised 29 March 1995; accepted 3 April 1995.

### Materials and methods

### *Source of tumour specimens*

The Office of Population Censuses and Surveys provided us with copies of the death certificates of all male butchers who had died of lung cancer in England and Wales during 1987 and 1988 ($n = 185$). By contacting the hospital where the subject had died or the doctor who had signed the death certificate, we sought archived histological material from the lung tumours that had caused the deaths. Where such material was available, we asked the pathology department concerned to send us six 10 μm formalin-fixed, paraffin-embedded sections, each cut with a fresh blade (some departments provided blocks from which similar sections were cut). In addition, we asked for details of how the specimen had been obtained, the histological diagnosis and, where it was convenient, for material from a control patient who was also a male with lung cancer and who was approximately the same age and diagnosed in the same year as the index case.

Suitable material was obtained from 40 butchers and 26 controls. The ages of these subjects, the source of the specimens and the histological diagnoses are summarised in Table I. Most specimens were from bronchial biopsies, and the most common diagnosis was squamous carcinoma. All patients with a non-pulmonary tissue source of tumour had histologically confirmed lung cancer.

### *Extraction of DNA from formalin-fixed paraffin-embedded tissues*

Sections of formalin-fixed and paraffin-embedded tissues were scraped from microscope slides or removed from the sterile tubes in which they were supplied, placed in 1.5 ml Eppendorf tubes and dewaxed by addition of 400 μl of xylene. The xylene was removed, 400 μl of 100% ethanol was added and the pellet was vortexed for 1 min, centrifuged for 5 min at 10 000 $g$ and the ethanol carefully pipetted off. The pellet was dried for 15 min and 100 μl of analar water was added to the pellet followed by 100 μl of paraffin oil to cover the solution. The tube was then boiled for 10 min. 10 μl volume of this solution contained a sufficient amount of DNA for the polymerase chain reaction (Shibata *et al.*, 1988).

In cases where DNA was not released by this procedure, extensive proteinase K digestion (100 μg ml$^{-1}$ final concentration in 5 mM Tris-HCl, 100 mM sodium chloride, 1 mM EDTA pH 8.0 and 0.5% sodium dodecyl sulphate) was carried out before phenol extraction, boiling and subsequent addition of the deproteinised solution to the PCR reaction.

*PCR conditions*

All procedures involving these sections were carried out in a laminar flow cabinet and a master stock of PCR reaction mix containing 100 mM Tris-HCl pH 8.3, 500 mM potassium chloride, 200 μM each of dATP, dCTP, dGTP and dTTP *Taq* DNA polymerase (NBL) (2.5 units per 100 μl reaction) and an optimised magnesium concentration was also prepared in a separate hood. For each virus-specific reaction, the master mixture also contained appropriate 20 bp primer pairs (Table II) at a final concentration of 0.2 μM. The master mix was aliquoted into 90 μl in separate Eppendorf tubes and overlaid by addition of paraffin oil.

Specific magnesium chloride concentrations were optimised for each of the PCR primer pairs used in the reaction, and these are listed together with the DNA sequences for these primers in Table II. Volumes of 10 μl of extracted tissues were mixed with 90 μl of reaction mixture containing the above components, and transferred to a Perkin Elmer Cetus model 480 thermal cycler and treated as follows: denaturation at 94°C for 3 min, followed by 30 cycles of 94°C for 1 min, 55°C for 1 min, 72°C for 1 min and a final extension at 72°C for 7 min.

After the amplification reaction, 5 μl from the 100 μl reaction mixture was loaded on to a 1.5% agarose gel containing 0.5 μg ml$^{-1}$ ethidium bromide and the presence of PCR products assessed by UV fluorescence after electrophoresis. To intensify questionable positives, the DNA was transferred from the agarose gel on to a Hybond N$^{+}$ membrane filter (Amersham International) following the manufacturer's instructions and hybridised in Amersham rapid hybridisation buffer for 1 h, again following the manufacturer's instructions, with a $^{32}$P end-labelled 40 bp oligonucleotide which was internal to the two PCR primer sites (Maitland and Lynas, 1991).

*Single-strand conformational polymorphism (SSCP) analysis for p53 mutation*

PCR primers spanning exons 5, 7 and 8 are shown in Table II. A separate 10 μl sample from each of the extracted sections of lung cancer was subjected to PCR under standard conditions with these primers and all the products again separated by gel electrophoresis. From positive electrophoresis samples 5 μl was taken and reamplified for a further ten cycles in a PCR reaction mixture depleted in unlabelled dCTP and containing 2.5 μ Ci of [$^{32}$P]dCTP (Amersham code no. PB 10219). The PCR reaction was terminated after ten cycles and the 1 μl of the reaction mixture (from a total of 25 μl) was mixed with 9 μl of sample buffer (0.1% SDS, 10 mM EDTA). Five microlitres of this mixture was then mixed with an equal volume of formamide loading dye (95% formamide, 20 mM EDTA, 0.5% bromophenol blue and 0.05% xylene cyanol). The samples were heated to 85°C for 5 min, chilled on ice and 4-5 μl was then loaded on to the SSCP gel.

*SCCP gel analysis*

The native polyacrylamide gel analysis for single-strand conformational polymorphism analysis was carried out essentially as described by Hayashi (1991). Initial results were obtained using 5% polyacrylamide gels containing 10% glycerol and 1 × TB buffer (89 mM Tris-borate, 2 mM

**Table I**

| | *Butchers* | *Controls* |
|---|---|---|
| Number of subjects | 40 | 26 |
| Age (years) | | |
| Mean | 65.2 | 66.4 |
| Range | 43-73 | 48-76 |
| Source of specimen (no. of subjects) | | |
| Bronchial biopsy | 19 | 16 |
| Lung biopsy | 4 | 1 |
| Surgical resection | 7 | 4 |
| Autopsy | 4 | 4 |
| Pleural biopsy | 1 | 0 |
| Mediastinal biopsy | 1 | 0 |
| Liver biopsy | 2 | 0 |
| Tonsillar biopsy | 1 | 0 |
| Unknown | 1 | 1 |
| Histological diagnosis (no. of subjects) | | |
| Squamous carcinoma | 19 | 14 |
| Adenocarcinoma | 2 | 2 |
| Oat cell carcinoma | 1 | 3 |
| Other carcinoma | 5 | 0 |
| Unavailable | 13 | 7 |

**Table II** Sequences of oligonucleotide primers

| *Primer type* | *Sequence 5′-3′* | *Location* | *Optimal Mg$^{++}$ (mM)* | *Size of amplification product (bp)* |
|---|---|---|---|---|
| HGPRT (A) | CTTGCTGGTGAAAAGGACCC | Exon 7-exon 8 | | |
| HGPRT (B) | GTCAAGGGCATATCCTACAA | Exon 7-exon 8 | 4.5 | 267 |
| p53 exon 5 (L) | CCTGCCCTCAACAAGATGTT | 596-616 | | |
| p53 exon 5 (R) | CCTCACAACCTCCGTCATGT | 716-736 | 1.5 | 140 |
| p53 exon 7 (L) | GGCTCTGACTGTACCACCAT | 890-910 | | |
| p53 exon 7 (R) | GGAGTCTTCCAGTGTGATGA | 974-994 | 1.5 | 101 |
| p53 exon 8 (L) | ATCTACTGGGACGGAACAGC | 1002-1122 | | |
| p53 exon 8 (R) | GAGAGGAGCTGGTGTTGTTG | 1140-1160 | 4.5 | 249 |
| Pan HPV (L) | TGGTACAATGGGCATATGAT | E1 | | |
| Pan HPV (R) | AATGGCTTTTGGAATTTACA | E1 | 3.5 | 444 |
| HPV 7 (L) | AATTACTTATGACAATCCTG | L2 | | |
| HPV 7 (R) | GACGTGTATATATGGTTCAC | L2 | 4.5 | 171 |
| HPV 6b (L) | GCTAATTCGGTGCTACCTGT | E6 gene | | |
| HPV 6b (R) | CTGGACAACATGCATGGAAG | E6 gene | 1.5 | 140 |
| HPV 11 (L) | CGCAGAGATATATGCAATATG | E6 gene | | |
| HPV 11 (R) | AGTTCTAAGCAACAGGCACA | E6 gene | 4.5 | 90 |
| HPV 16 (L) | TCAAAAGCCACTGTGTCCTG | E6 gene | | |
| HPV 16 (R) | CGTGTTCTTGATGATCTGCA | E6 gene | 3.5 | 120 |
| HPV 18 (L) | ACCTTAATGAAAAACCACGA | E6 gene | | |
| HPV 18 (R) | CGTCGTTGGAGTCGTTCCTG | E6 gene | 1.5 | 100 |

L, left (5′) primer; R, right (3′) primer; HGPRT, human hypoxanthine guanine phosphoribosyl transferase. Pan-HPV is GP1/GP2 (Van den Brule, 1993).

EDTA pH 8.1). Electrophoresis was carried out at 40 W, 18°C. Some of the data were obtained using 5% glycerol and 0.5 × TB as described by Orita *et al.* (1989). After electrophoresis the native polyacrylamide gel was dried and subjected to autoradiography at −70°C with Dupont Cronex Lightning plus intensifying screens for 16–48 h.

*DNA sequencing*

Since, at the start of this work, only very limited DNA sequence information was available for HPV 7, we obtained the reference clone from E-M de Villiers and H zur Hausen, German Cancer Research Centre, Heidelberg (Oltersdorf *et al.*, 1986), and sequenced portions of it as a series of overlapping fragments (approximately 2 kb in total) initiating from primers within the pBR322 cloning vector using *Taq* cycle sequencing (US Biochemical). These sequences encoded the HPV 7 E1 and L2 genes (by comparison with HPV 11) and were used as the basis for HPV 7 PCR primer design, with the Primer Designer program (Scientific and Technical Software, State Line, PA, USA). The fidelity of these sequences and their coding potential has recently been confirmed by Dr H Delius, who has completely sequenced the same HPV 7 clone (Delius and Hoffman, 1994). *Taq* cycle sequencing was also used to confirm the presence of p53 mutations detected by SSCP.

### Results

*Detection of human papillomaviruses in tumours*

The 66 tumour samples were examined for the presence of various types of HPV DNA using PCR. The E1 consensus primers shown in Table II produced an amplification product of 444 bp. Six samples (three from butchers and three from controls) were shown to be positive with these E1 primers, an example of which is shown in Figure 1a and the complete data summarised in Table III. We subsequently employed primers specific for HPV types 6, 7, 11, 16 and 18 on these samples. DNA from the oncogenic HPV 16 was demonstrated in one control lung cancer, while benign type HPV DNA was found in three subjects: one butcher and one control with HPV 11, and a further control with both HPV 6 and 11 (Figure 1b). Two butchers positive for the consensus E1 primer (van den Brule *et al.*, 1993) showed no PCR products when examined by the type-specific primer method, which suggested the presence of an unusual or new HPV type in these samples. All of the samples were tested with HPV 7-specific primers which had previously been shown (Keefe *et al.*, 1994*a*) to react specifically with HPV type 7 in hand warts from butchers, but HPV 7 DNA was not found in any of the tumours (Figure 1c). The ability of all of the DNA extracts to act as a template for PCR was verified by a positive result with primers for the human HPRT gene (data not shown) and by a positive result (Table III) for the p53 exon-specific primers.

**Figure 1** Detection of HPV DNA in lung cancer biopsy material by consensus and type-specific PCR. (**a**) Consensus primer-positive results (lanes 5, 11 and 16 contain the 444 bp product indicated by ●). (**b**) HPV 11 type-specific primers with positive signals in lanes 2, 3 and 5 (90 bp product indicated by ●). (**c**) HPV 7-specific primers showing no positive results in the lung samples (171 bp product indicated by ●). Size markers are (**a**) Øx174 RF DNA digested with *Hinf*I and (**b** and **c**) 100 bp ladder.

**Table III** Summary of HPV DNA and p53 mutation detection in lung cancers

| | *Butchers* | | *Non-butchers* | |
|---|---|---|---|---|
| *Confirmed histology* | *HPV DNA* | *p53 mutation* | *HPV DNA* | *p53 mutation* |
| Squamous cell carcinoma | 2/21 | 3/21 | 1/12 | 2/12 |
| Adenocarcinoma | 0/2 | 0/2 | 0/2 | 0/2 |
| Oat cell carcinoma | 0/1 | 0/1 | 0/3 | 0/3 |
| Non-small cell carcinoma | 0/1 | 0/1 | – | – |
| Unconfirmed carcinoma (mostly SCC) | 1/15 | 4/15 | 2/9 | 0/9 |
| Totals | 3/40 | 7/40 | 3/26 | 2/26 |

**Figure 2** SSCP detection of point mutations in p53 gene exon 5. Arrows indicate the position of mutant strand mobilities in lane 3, which also contains a copy of the normal allele. The + indicates DNA from normal human epithelium.

*Detection of p53 mutations in lung cancers*

We also tested for p53 mutation, which has been suggested as an important mutagenic step in the development of lung cancers. Specific primers for exons 5, 7 and 8, which should encompass approximately 80% of p53 mutations detected to date in lung cancers (Hollstein *et al.*, 1994), were used in the PCR amplification. The average frequency of p53 mutations in exon 6, the only other significant hotspot, is about 13% in lung cancers. Because of shortage of material and the requirement to use the same samples for both HPV detection and p53 detection, the total number of samples amplified for all the p53 primers in this group was only 46.

After PCR amplification of exons 5, 7 and 8 of p53, abnormal SSCP mobilities, implying gene mutations, were observed in nine (six squamous carcinomas and three tumours for which no detailed histology report was available). Seven mutations were found in exon 5, two in exon 7 and none in the region containing exon 8 (summarised in Table III). An example of the p53 amplification and SSCP is shown in Figure 2. This shows a clear shift in the pattern of fragment mobility on the SSCP gel after denaturation and refolding of the DNA fragments. All of the potential p53 mutations were confirmed by direct DNA sequencing of the PCR products, and in all cases substantial signal from the normal allele was also present, which indicated that most of the tumours were mutant/normal heterozygotes. No individual tumour sample in which a p53 mutation was detected (9/46) also contained HPV DNA (6/46). Given the low positive rates, however, this is not statistically significant.

### Discussion

This study provides no evidence that HPV 7 or the other HPVs tested are important causes of lung cancer in butchers. The initial screen, using a consensus primer pair, showed that only a small proportion of lung tumours contained HPV DNA. This is entirely in agreement with previous work (Bejui-Thiviolet *et al.*, 1990) and another recent PCR-based study of lung cancers from the general population (Shamanin *et al.*, 1994). The PCR method that we used is sensitive enough to detect between one and five viral genomes in a large excess of cellular DNA, but only six cases of HPV infection were found, and further studies with type-specific primers detected no evidence of HPV 7. HPV 7 is so common in butchers that most of the butchers studied are likely to have been exposed to the virus. In a recent survey, 34% of a butcher population had a history of wart infection (Keefe *et al.*, 1994*a*). From this we would estimate that some 15–25% of them would have had HPV 7-associated hand warts at some stage in their working lives. Moreover, if HPV 7 were the explanation for the more than 50% excess of lung cancer that has been reported in butchers, then this virus would have been expected in at least 13 of the 40 butchers' tumours examined.

It remains possible that HPV 7 could initiate the carcinogenic process in a 'hit and run' fashion, and not be present or expressed in the resulting tumour. This mechanism has been proposed in bovine alimentary tract carcinomas, in which BPV4 is detected in non-malignant papillomas, whereas viral DNA is completely absent in the tumours which develop as a consequence of the action of a chemical co-carcinogen (Campo *et al.*, 1985). Since we have not studied putative precursor lesions, we cannot eliminate this possibility for HPV 7. Also, since our PCR primers for HPV 7 detection were located in the L2 open reading frame (ORF), a region which is frequently eliminated from high-risk HPV types in cervical cancer upon integration into the cell chromosome, we cannot eliminate the possibility that only the HPV 7 E6 and E7 ORFs for example have been retained in the butchers' tumours. However, in cervical tumour biopsies, between 30% and 90% of cases contained episomal full-length HPV genomes along with the integrated viral genes (Fuchs *et al.*, 1989; Matsukara *et al.*, 1989; Kristiansen *et al.*, 1994). If the same situation were to apply in the butchers' tumours, then at least a proportion of the carcinomas would have retained the L2 ORF of HPV 7, were it an important aetiological agent.

Mutations in the p53 gene were detected by SSCP analysis in 22% of the butchers and 9.5% of the controls. Previous studies suggest that at least 50% of lung cancers have p53 mutations (Chiba *et al.*, 1990; Charles *et al.*, 1991; Takahashi *et al.*, 1992). Given that SSCP is capable of detecting approximately 80% of potential mutations (Hayashi, 1991) and that the three exons chosen should account for up to 80% of those discovered to date in the p53 genome in lung cancers (Caron de Fromental and Soussi, 1992; Hollstein *et al.*, 1994), the method we used is likely to detect about 60% of any p53 mutations that are present (Dunn *et al.*, 1993), so about 30% of the samples should have been affected, rather more than we detected. The low frequency could be accounted for either by an abnormally high prevalence of butcher-specific mutations outwith exons 5, 7 and 8 (for example in exon 6, since this exon was not included in the screen) or by a targeted mutation in a base for which SSCP is ineffective at mutant detection, or simply by the fact that p53 mutations are less frequent in this group of lung cancers. Perhaps the most likely explanation is dilution of the mutant allele with an excess of normal cells in the tissue sections.

Urogenital cancers associated with HPV infection usually contain wild-type p53 rather than mutant p53 (Crook *et al.*, 1991) although not inevitably so (Busby-Earle *et al.*, 1994). In our study no HPV DNA-positive carcinoma contained a detectable p53 mutation. The p53 protein can be bound and its degradation accelerated by the HPV E6 protein from high-risk HPV types (Scheffner *et al.*, 1993), but the low level of HPV detection in this study indicates that HPV infection is probably not the reason for the lower frequency of p53 mutation observed. Interestingly, both p53 mutation and HPV detection were confined to squamous cell carcinoma and were completely absent from samples of normal lung (data not shown) and other histological types of tumour, although the numbers of non-squamous cell carcinomas were small.

If HPV 7 is not commonly associated with lung cancer in butchers then how can the apparent excess of cases be explained? In a recent study (Keefe *et al.*, 1994*a*, *b*) we found a 50% prevalence of smoking in a sample of 486 butchers. This is higher than in the general population of Britain (average around 33%), and high rates of smoking might account for the raised incidence of lung cancer. Alternatively, there could be a chemical carcinogen in the workplace (Johnson, 1994). Possible candidates include the polycyclic aromatic hydrocarbons produced when meat is smoked, nitrites used in the preservation of some meats and fumes from plastic used to wrap meat. However, current epidemiological evidence does not point strongly to any of those as the culprit.

**Acknowledgements**
This work was carried out by AAA as part of his PhD studies, supported by the Royal Kingdom of Saudi Arabia. We wish to thank both the Yorkshire Cancer Research Campaign and the Medical Research Council, OPCS, for help in identifying lung cancer cases in butchers, Jo Birch for typing of the manuscript and Meg Stark and Peter Crosby for photographic services.

### References

ADLER-STORTHZ K, NEWLAND J, TESSIN B, YENDALL W AND SHILLITOE E. (1986). Human papillomavirus type 2 DNA in oral verrucous carcinoma. *J. Oral Pathol.*, **15**, 472–475.

BEJUI-THIVOLET F, CHARDONNET Y AND PATRICOT LM. (1990). Human papillomavirus type 11 DNA in papillary squamous cell lung carcinoma. *Virchows. Arch.*, **417**, 457–461.

BENTON EC. (1994). Warts in butchers - a cause for concern. *Lancet*, **343**, 1114.

BUSBY-EARLE RMC, STEEL CM, WILLIAMS ARW, COHEN B AND BIRD CC. (1994). p53 mutations in cervical carcinogenesis: low frequency and lack of correlation with human papillomavirus status. *Br. J. Cancer*, **69**, 732-737.

CAMPO MS, MOAR MS, SARTIRANA ML, KENNEDY IM AND JARRETT WFH. (1985). The presence of bovine papillomavirus type 4 DNA is not required for the progression to or maintenance of the malignant state in papillomavirus associated carcinomas of the alimentary tract in cattle. *EMBO J.*, **4**, 1819-1825.

CAREY FA, SALTER DM, KERR KM AND LAMB D. (1990). An investigation into the role of human papillomavirus in endobronchial papillary squamous tumours. *Respir. Med.*, **84**, 445-447.

CARON DE FROMENTEL C AND SOUSSI T. (1992). TP53 tumour suppressor gene: a model for investigating human mutagenesis. *Genes Chrom. Cancer*, **4**, 1-15.

CHARLES H, HENSEL RH, SAKAGUCHI AY AND NAYLOR SL. (1991). Use of the single strand conformation polymorphism technique and PCR to detect p53 gene mutations in small cell lung cancer. *Oncogene*, **6**, 1067-1071.

CHIBA I, TAKAHASHI T, NAU MM, AMICO D AND CURIEL DT. (1990). Mutations in the p53 gene are frequent in primary, resected non-small cell lung cancer. *Oncogene*, **5**, 1603-1610.

COGGON D, PANNETT B, PIPPARD EC AND WINTER PD. (1989). Lung cancer in the meat industry. *Br. J. Indust. Med.*, **46**, 188-191.

CROOK T, WREDE D AND VOUSDEN KH. (1991). p53 point mutation in HPV negative human cervical carcinoma cell lines. *Oncogene*, **6**, 873-875.

DE PEUTER M, DE CLERCQ B AND MINNETTE A. (1977). An epidemiological survey of virus warts of the hands among butchers. *Br. J. Dermatol.*, **96**, 427-431.

DE VILLIERS E, WEIDAUER H, OTTO H AND ZUR HAUSEN H. (1985). Papillomavirus DNA in human tongue carcinomas. *Int. J. Cancer*, **36**, 575-578.

DELIUS H AND HOFFMAN B. (1994). Primer directed sequencing of human papillomavirus types. In *Current Topics in Microbiology and Immunology*, Vol. 186, Capron A, Compans RW, Cooper M *et al.*, (eds) pp. 13-31. Springer: Heidelberg.

DUNN JM, HASTRICH DJ, WEBB JCJ, MAITLAND NJ AND FARNDON JR. (1993). Correlation between p53 mutations and antibody staining in breast carcinoma. *Br. J. Surg.*, **80**, 1410-1412.

FUCHS P, GIRARDI F AND PFISTER H. (1989). Human papillomavirus type 16 in cervical cancers and in lymph nodes of cervical cancer patients: a diagnostic marker for early metastases? *Int. J. Cancer*, **43**, 41-44.

GREENSPAN D, DE VILLIERS EM, GREENSPAN JS, DE SOUZA YG AND ZUR HAUSEN H. (1988). Unusual HPV types in oral warts in association with HIV infection. *J. Oral Pathol.*, **17**, 482-487.

GUILLON L, SAHLI R, CHAUBERT P, MONNIER P, CUTTAT JF AND COSTA J. (1991). Squamous cell carcinoma of the lung in a nonsmoking, nonirradiated patient with juvenile laryngotracheal papillomatosis: evidence of human papillomavirus-11 DNA in both carcinoma and papillomas. *Am. J. Surg. Pathol.*, **15**, 891-898.

HAYASHI K. (1991). PCR-SCCP: a simple and sensitive method for detection of mutations in the genomic DNA. *PCR Methods Applic.*, **1**, 34-38.

HOLLSTEIN M, RICE K, GREENBLATT MS, SOUSSI T, FUCHS R, SORLIE T, HOVIG E, SMITH-SORENSEN B, MONTESANO R AND HARRIS CC. (1994). Database of p53 gene somatic mutations in human tumours. *Nucleic Acids Res.*, **22**, 3551-3555.

JOHNSON ES. (1994). Poultry, oncogenic retroviruses and humans. *Cancer Detect. Prev.*, **18**, 9-30.

KEEFE M, AL-GHAMDI AA, COGGON D, MAITLAND NJ, EGGER P, KEEFE CJ, CAREY A AND SANDERS CM. (1994*a*). Cutaneous warts in butchers. *Br. J. Dermatol.*, **130**, 9-14.

KEEFE M, AL-GHAMDI AA, MAITLAND NJ, SANDERS CM, COGGON D, CAREY A, EGGER P AND KEEFE C. (1994*b*). Butchers' warts: no evidence for person to person transmission. *Br. J. Dermatol.*, **130**, 15-17.

KRISTIANSEN E, JENKINS A AND HOLM R. (1994). Co-existence of episomal and integrated HPV16 DNA in squamous cell carcinoma of the cervix. *J. Clin. Pathol.*, **47**, 253-256.

LEVI JE, DELCELO R, ALBERTI VN *et al.* (1989). Papillomavirus DNA in respiratory papillomatosis detected by in situ hybridization and the polymerase chain reaction. *Am. J. Pathol.*, **135**, 1179-1184.

LITT J. (1969). Warts in meat-cutters. *Arch. Dermatol.*, **100**, 773.

MAITLAND NJ AND LYNAS C. (1991). The detection of latent virus infection by polymerase chain reaction. In *Methods in Molecular Biology*, Vol. 9, *Protocols in Human Molecular Genetics*. Mathew C. (ed.) pp. 347-364. Humana Press: Clifton, NJ.

MATSUKARA T, KOI S AND SUGASE M. (1989). Both episomal and integrated forms of human papillomavirus type 16 are involved in invasive cervical cancers. *Virology*, **172**, 63-72.

MELCHERS W, DE MARE S, KUITERT E, GALAMA J, WALBOOMERS J AND VAN DEN BRULE AJC. (1993). Human papillomavirus and cutaneous warts in meat handlers. *J. Clin. Microbiol.*, **31**, 2547-2549.

MOUNTS P AND SHAH K. (1984). Respiratory papillomatosis: etiological relation to genital tract papilloma viruses. *Prog. Med. Virol.*, **29**, 90-114.

OLTERSDORF T, CAMPO MS, FAVRE M, DARTMANN K AND GISSMANN L. (1986). Molecular cloning and characterisation of human papillomavirus type 7 DNA. *Virology*, **149**, 247-250.

ORITA M, YOUICHI S, SEIYA T AND HAYASHI K. (1989). Rapid sensitive detection of point mutations and DNA polymorphisms using the polymerase chain reaction. *Genomics*, **5**, 874-879.

ORTH G, BREITBURD F, FAVRE M AND CROSSANT O. (1977). Cell proliferation. In *Origins of Human Cancer*, Hiatt H *et al.* (eds). p. 1043. Cold Spring Harbor Laboratory Press: USA.

ORTH G, JABLONSKA S, FAVRE M, CROISSANT Ó, OBALEK S, JARZABEK-CHORZELSKA M AND JIBARD N. (1981). Identification of papillomaviruses in butchers' warts. *J. Invest. Dermatol.*, **76**, 97-102.

OSTROW R, KRYZYZEK R, PASS F AND FARAS A. (1981). Identification of a novel human papillomavirus in cutaneous warts of meathandlers. *Virology*, **108**, 21-27.

RUDLINGER R, BUNNEY MH, GROP R AND HUNTER JAA. (1989). Warts in fish handlers. *Br. J. Dermatol.*, **120**, 375-381.

SCHEFFNER M, HUIBREGTSE JM, VIERSTRA RD AND HOWLEY PM. (1993). The HPV16 E6 and E6-AP complex functions as a ubiquitin-protein ligase in the ubiquitination of p53. *Cell*, **75**, 495-505.

SHAMANIN V, DELIUS H AND DE VILLIERS E-M. (1994). Development of a broad spectrum PCR assay for papillomaviruses and its application in screening lung cancer biopsies. *J. Gen. Virol.*, **75**, 1149-1156.

SHIBATA DK, ARNHEIM N AND MARTIN J. (1988). Detection of human papilloma virus in paraffin-embedded tissue using the polymerase chain reaction. *J. Exp. Med.*, **167**, 225-230.

SYRJANEN S, VON KROGH G, KELLOASKI J AND SYRJANEN K. (1989). Two different human papillomavirus types associated with oral mucosal lesions in an HIV-seropositive man. *J. Oral Pathol. Med.*, **18**, 366-370.

TAKAHASHI T, SUZUKI H, HIDA T, SEKIDO Y, ARIYOSHI Y AND UEDA R. (1992). p53 mutations in non-small cell lung cancer in Japan: association between mutations and smoking. *Cancer Res.*, **52**, 734-736.

VAN DEN BRULE AJC, SNIJDERS PJF, MEIJER CJLM AND WALBOOMERS JMM. (1993). PCR-based detection of genital HPV genotypes: an update and future perspectives. *Papillomavirus Rep.*, **4**, 95-99.

VAN RANST M, KAPLAN JB AND BURK RD. (1992). Phylogenetic classification of human papillomaviruses: correlation with clinical manifestations. *J. Gen. Virol.*, **73**, 2653-2660.

YOUSEM SA, OHORI NP AND SONMEZ-ALPAN E. (1992). Occurrence of human papillomavirus DNA in primary lung neoplasms. *Cancer*, **69**, 693-697.

ZUR HAUSEN H. (1988). Papillomaviruses in human cancers. *Mol. Carcinogen.*, **1**, 147-150.